# Management of pulmonary arteriovenous malformations involves additional factors aside from the diameter of feeding arteries: a 3-year case-case retrospective analysis

**DOI:** 10.1186/s12931-022-02030-9

**Published:** 2022-04-30

**Authors:** Xu Ma, Ling-Ling Li, Dong Yu, Bing Jie, Sen Jiang

**Affiliations:** Department of Radiology, Shanghai Pulmonary Hospital, School of Medicine, Tongji University, No. 507 Zhengmin Road, Shanghai, 200433 China

**Keywords:** Pulmonary arteriovenous malformation, Hereditary haemorrhagic telangiectasia, Hypoxia, Transient ischaemic attack, Embolisation

## Abstract

**Background:**

Pulmonary arteriovenous malformations (PAVMs) are rare but cause various manifestations. Although the diameter of feeding arteries has been linked to treatment decisions, relationships among the characteristics of PAVMs, clinical symptoms, and treatment effect remain unclear. The present study was performed to investigate how collective characteristics of PAVMs relate to clinical symptoms and to provide proper treatment recommendations for patients with PAVMs.

**Methods:**

We retrospectively analysed 838,447 patients’ radiographic data and medical records from January 2018 to December 2020. Patients were included if a PAVM was radiographically detected for the first time in our hospital. Ordered multivariable logistic regression and hierarchical multiple regression were performed to analyse the relationships between characteristics of PAVMs and various clinical symptoms. We investigated the management of PAVMs in four tertiary university hospitals.

**Results:**

Detection rate of PAVMs was 0.025% (207/838,447), and 37.6% of patients (78/207) also had hereditary haemorrhagic telangiectasia. Eight patients were diagnosed with bilateral diffuse PAVMs. Two hundred thirty-six lesions were detected in 199 patients, and the mean diameter of the feeding artery was 4.13 ± 1.92 mm. Most PAVMs were the simple type and located in the peripheral pulmonary area. In total, 34.3% of patients (71/207) were symptom-free; remaining patients showed various manifestations, and respiratory symptoms were most common (dyspnoea on exertion, 47.8%). The diameter of the feeding artery and the type and the number of PAVMs were correlated with hypoxaemia (P < 0.001, P < 0.001, and P = 0.037, respectively). The collective characteristics of PAVMs were not related to the severity of central nervous system symptoms (largest diameter of feeding artery, P = 0.8; largest diameter of sac, P = 0.42; number of PAVMs, P = 0.35; type of PAVMs, P = 0.99). Various symptoms were greatly relieved after treatment. The hospital investigation showed that management of PAVMs was not generally appreciated in clinical practice.

**Conclusions:**

Our study revealed a low detection rate of PAVMs and a low degree of association with hereditary haemorrhagic telangiectasia in the general population. Considering the connection between collective characteristics of PAVMs and various clinical symptoms, clinicians should consider the type and number of PAVMs, the largest diameter of the feeding artery, and clinical symptoms when managing patients with PAVMs.

## Background

Pulmonary arteriovenous malformations (PAVMs) are characterised by the presence of a direct pulmonary artery–vein shunt [1]. Most PAVMs are congenital and are closely associated with hereditary haemorrhagic telangiectasia (HHT) [2–4]. PAVMs have a low incidence worldwide and usually produce no obvious clinical manifestations [5–7]. However, serious complications such as stroke, brain abscesses, haemoptysis, haemothorax, and hypoxaemia sometimes occur because of the absence of a capillary bed [8–12]. Endovascular embolisation is recommended as the first-line treatment for PAVMs with a feeding artery of ≥ 3 mm in diameter [13, 14].

Recent research has mainly focused on the correlation between PAVMs and HHT and on treatment methods. Serious complications of PAVMs have also been described in some case reports [15–17]. However, the populations of previous studies comprised patients with HHT, and the correlation between the characteristics of PAVMs and associated clinical symptoms has not been specifically analysed.

The present study was performed to identify the characteristics of PAVMs in a large general population-based study cohort. By assessing multiple characteristics of PAVMs that influence various clinical symptoms, we aimed to provide proper suggestions for the management of PAVMs.

## Methods

### Clinical characteristics of patients with PAVMs

In this retrospective observational study, we reviewed the radiographic reports of 838,447 patients who presented to our hospital from January 2018 to December 2020. The review group consists of five radiologists with experience in radiodiagnosis and embolisation. Firstly, two radiologists with over 7 years of experience (X.M., L.L.L.) carried out the reports review process, and all the reports regarding PAVMs and pulmonary vascular abnormalities were included for further review. Then three radiologists with over 15 years of experience (D.Y., B.J., S.J.) performed the imaging review. PAVMs were confirmed by multi-slice spiral computed tomography (MSCT) (slice thickness, 1 mm; slice gap, 1 mm) or multi-slice spiral CT angiography (CTA) (slice thickness, 0.625 mm; slice gap, 0.625 mm). All detected PAVMs were classified as the simple type or the complex type based on the MSCT or CTA findings as described in previous studies [2, 18, 19]. The complex type included the following two subtypes: the diffuse subtype (involvement of a large part of the parenchyma) and the telangiectatic subtype (multiple microscopic vascular networks) [20, 21]. All enrolled patients were divided into different groups according to the number of PAVMs, and all detected PAVMs were grouped based on their location in the lung. The largest diameter of the feeding artery and the largest diameter of the sac of each PAVM was recorded based on the MSCT or CTA findings.

The clinical data, respiratory symptoms, central nervous system symptoms, and treatment regimens of all enrolled patients were obtained from the medical records and telephone follow-ups. The clinical diagnosis of HHT was confirmed according to the Curaçao criteria: (1) spontaneous epistaxis; (2) multiple telangiectasias of the body surface; (3) visceral lesions in the gastrointestinal mucosa, liver, or brain; and (4) first-degree relatives with HHT. Patients who met two criteria had a possible clinical diagnosis of HHT, and those who met three criteria had a definite diagnosis of HHT [8, 22]. Among patients who underwent embolisation, CTA was performed in the first month after embolisation, and MSCT scans were regularly performed if there was no evidence of PAVM recurrence as shown by MSCT.

### Current treatment status of PAVMs in clinical practice

We performed a questionnaire-based investigation in four tertiary university hospitals. This investigation involved the pathogenesis, related complications, and treatment strategy of PAVMs. The results were obtained from 121 doctors in the departments of respiration, thoracic surgery, radiology, oncology, intervention, and emergency in 4 different tertiary university hospitals.

### Statistical analysis

All results are expressed as mean ± standard deviation or frequency (percentage). The statistical analysis was performed using SPSS version 19.0 (IBM Corp., Armonk, NY, USA). A P-value of < 0.05 was considered statistically significant.

Valid results of blood gas analyses (exclusion criteria: blood samples obtained during oxygen therapy or massive haemoptysis) were obtained from 64 enrolled patients, and hierarchical multiple regression analysis was used to determine the relationship of multiple factors (collective characteristics of PAVMs, age, sex, and other lung diseases) with the oxygen pressure. Simple linear regression and variable correlation scatter plots were used to analyse the correlation between the diameter of the feeding artery and oxygen pressure. Paired-sample t-tests were applied to compare the results of the blood gas analysis before and after treatment of PAVMs in 24 enrolled patients.

The severity of central nervous system symptoms was classified on a scale of 1 to 4 (level 1, none; level 2, headache or dizziness; level 3, transient ischaemic attack [TIA]; and level 4, stroke or brain abscess). Ordered multivariable logistic regression analysis was performed to analyse the relationship between multiple factors (collective characteristics of PAVMs, age, and sex) and central nervous system symptoms in all enrolled patients. Differences of the largest diameter of the feeding artery in different levels were compared with One-way ANOVA analysis.

## Results

### Baseline characteristics of patients and detected PAVMs

Among 838,447 patients, 207 patients were enrolled in the present study, and the detection rate of PAVMs was 0.025%. The baseline characteristics of all enrolled patients are shown in Table 1. In total, 77.78% of the enrolled patients were female. The patients’ mean age was 53 ± 14.2 years (range 14–87 years). A clinical diagnosis of definite or possible HHT was made in 37.7% of patients (78/207), and 73.1% of these 78 patients were female. Eight patients with HHT had bilateral diffuse multiple PAVMs (n > 10), including multiple telangiectatic lesions, simple lesions, and complex lesions. One hundred twenty-eight patients chose to undergo regular follow-up. Endovascular embolisation was performed in 76 patients, and 4 patients chose video-assisted thoracoscopic surgery. One patient diagnosed with a diffuse PAVM received embolisation because of intermittent haemoptysis. Massive haemoptysis recurred 2 days after embolisation, and the patient then underwent video-assisted thoracoscopic surgery.

**Table 1:** Baseline characteristics of patients diagnosed with PAVMs

Parameter	Value
Sex	
Male	46(22.22%)
Female	161(77.78%)
Age	
Average	53 ± 14.2
Range	14–87
HHT(F/M)	
Negative	129(105/24)
Possible	29(23/6)
Definite	49(34/15)
Methods of examination	
MSCT	103
CTA	104
Type of number (HHT/None)	
Solitary	181 (59/122)
Unilateral multiple	5 (3/2)
Bilateral multiple (≤ 10)	13 (8/5)
Bilateral diffuse multiple (> 10)	8 (8/0)
Treatment of PAVMs	
Follow-up observations	128 (61.8%)
Embolization	76 (36.7%)
Thoracoscope	4 (2%)
Embolization and thoracoscope	1 (0.5%)

Data are presented as mean ± standard deviation or frequency (percentage). *PAVMs* pulmonary arteriovenous malformations, *HHT* hereditary haemorrhagic telangiectasia, *M* male, *F* female, *MSCT* multi-slice spiral computed tomography, *CTA* computed tomography angiography

Table 2 shows the patients’ clinical manifestations. Seventy-one (34.3%) of 207 patients were asymptomatic. Ninety-nine (47.8%) enrolled patients exhibited dyspnoea on exertion. Forty-three patients (20.8%) exhibited serious central nervous system manifestations (TIA, stroke, or brain abscess), and their mean age was 45.58 ± 15.23 years (range 14–83 years; median 47 years).

**Table 2:** Clinical manifestations of patients with PAVMs

Respiratory system related	n (%)	Central nervous system related	n (%)	Other complains	n (%)
Chest distress	84 (40.6)	Headache	42 (20.3)	Symptom-free	71 (34.3)
Chest pain	39 (18.8)	Dizziness	33 (15.9)		
Dyspnoea	66 (31.9)	TIA	34 (16.4)		
Dyspnoea on exertion	99 (47.8)	Stroke	8 (3.9)		
Cyanosis	24 (11.6)	Brain abscess	1 (0.5)		
Clubbing finger	16 (7.7)				
Hemoptysis	28 (13.5)				

Data are presented as frequency (percentage). *PAVMs* pulmonary arteriovenous malformations, *TIA* transient ischaemic attack

**Fig. 1:**
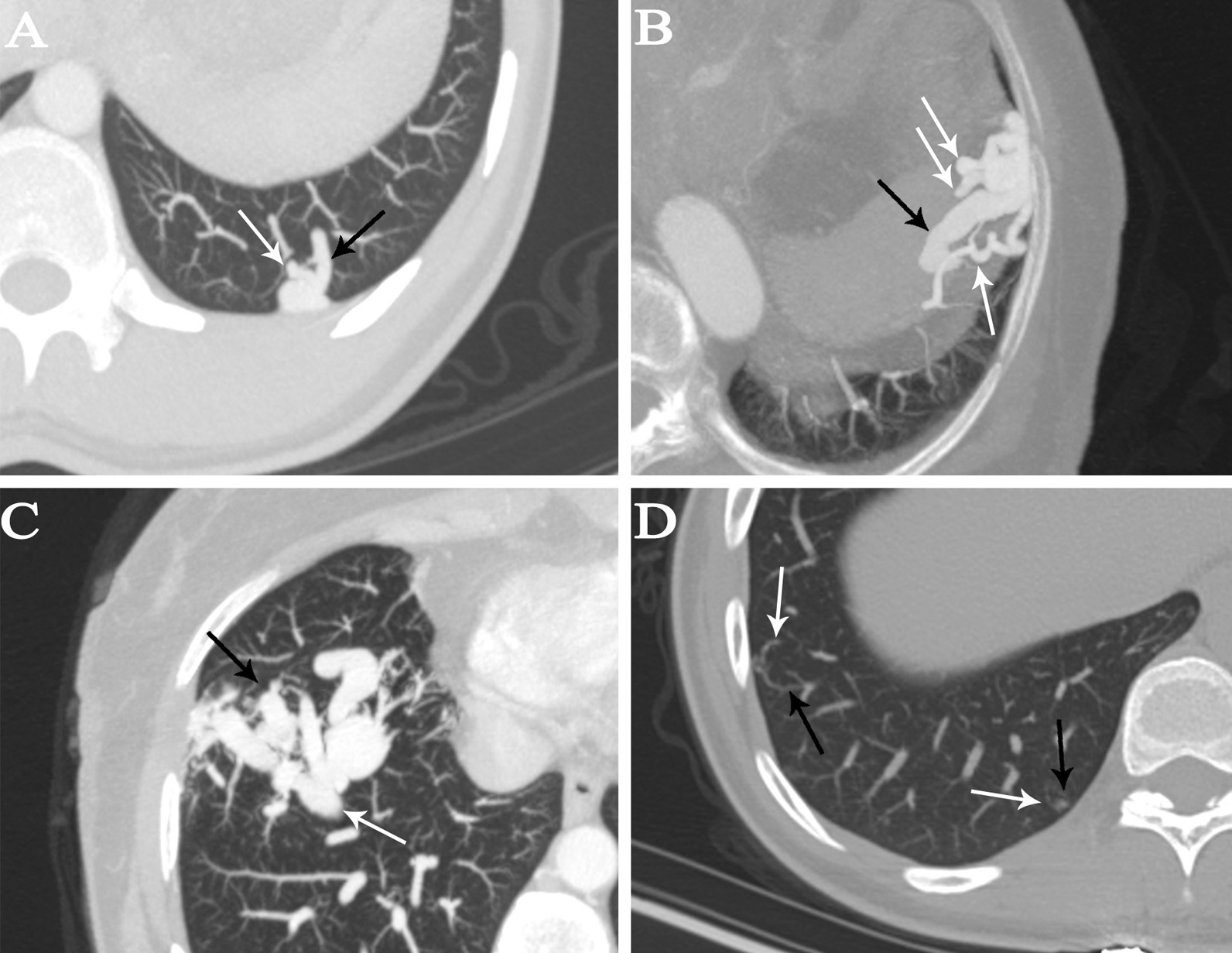
All types of PAVMs were detected by MSCT and CTA. **A** The simple type of PAVM consisted of one feeding artery (white arrow) and one draining vein (black arrow). **B** Three feeding arteries (white arrows) and one enlarged draining vein (black arrow) were detected in one complex PAVM. **C** One diffuse PAVM had multiple feeding arteries involving a large part of the parenchyma (black arrow) and one enlarged draining vein (white arrow). **D** Telangiectatic PAVMs with one feeding artery (white arrows) and one draining vein (black arrows) were detected by MSCT. *PAVMs* pulmonary arteriovenous malformations, *MSCT* multi-slice spiral computed tomography, *CTA* computed tomography angiography

Two hundred thirty-six PAVMs were detected in 199 patients, excluding 8 patients with bilateral diffuse multiple PAVMs (n > 10). Of the 236 lesions, 209 (88.6%) were located in the peripheral pulmonary area. The simple type was the most common type among these 236 PAVMs (88.6%). Two diffuse PAVMs were detected in two patients, and all six telangiectatic PAVMs were detected in a single patient with HHT (Fig. 1). The mean largest diameter of the feeding artery and mean largest diameter of the sac was 4.13 ± 1.92 mm and 10.31 ± 7.48 mm, respectively (Table 3).

**Table 3:** Characteristics of PAVMs in 199 patients

Characters	Value(%)
Total number of PAVM	236
Location	
Subpleural	177(75%)
Outer1/3	32 (13.6%)
Inner	27 (11.4%)
Type of PAVM	
Simple type	209 (88.6%)
Complex type	19 (8.1%)
Diffuse subtype	2 (0.8%)
Telangiectatic subtype	6 (2.5%)
Largest diameter of the feeding artery (mm)	4.13 ± 1.92
Largest diameter of the sac (mm)	10.31 ± 7.48

Data are presented as mean ± standard deviation or frequency (percentage). *PAVMs* pulmonary arteriovenous malformations

**Fig. 2:**
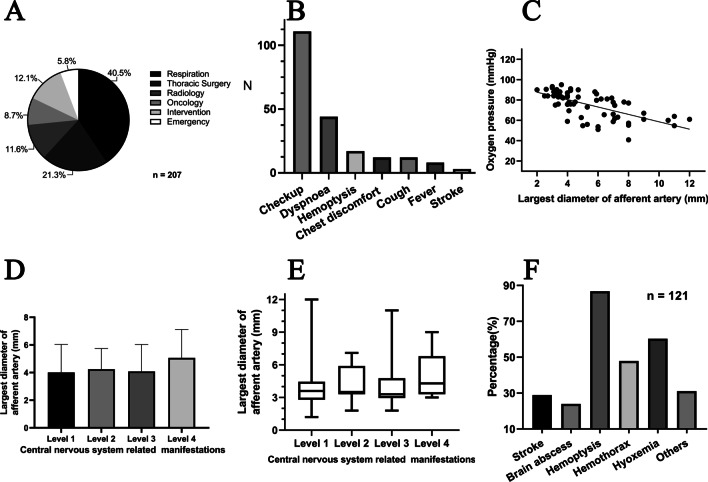
**A** Distribution of the departments that patients with PAVMs visited for consultation. **B** Different reasons for the 207 patients’ first visit to the hospital: health check-up (53.6%), dyspnoea (21.3%), haemoptysis (8.2%), chest discomfort (5.8%), cough (5.8%), fever (3.9%), and stroke (1.4%). **C** The diameter of the feeding artery had a significant linear correlation with the oxygen pressure prior to any treatments (P < 0.0001, R^2^ = 0.4308, y = − 3.639x + 94.988). **D** There were no significant differences in the largest diameter of the feeding artery among patients with different levels of central nervous system symptoms (P = 0.44). **E** The range of the largest diameter of the feeding artery was similar among all levels (P = 0.95). **F** The graph shows the survey results of PAVM-related serious complications as reported by the investigated doctors; 31.4% of doctors (38/121) did not fully understand all the serious complications. *PAVMs* pulmonary arteriovenous malformations

Most of the enrolled patients visited the department of respiration (40.5%) or thoracic surgery (21.3%) (Fig. 2A). One hundred eleven patients (53.6%) were first diagnosed with PAVMs during a health check-up. The chief complaint in 44 patients (21.3%) was various degrees of dyspnoea at their first visit, and the main causes of hospital visits were different respiratory manifestations (44.9%, 93/207) (Fig. 2B).

### Associations between collective characteristics of PAVMs and hypoxaemia

The results of the hierarchical multiple regression analysis of the relationship between multiple factors and hypoxaemia are shown in Table 4. The largest diameter of the feeding artery, type and number of PAVMs, and other lung diseases were closely associated with hypoxaemia (P < 0.001, P < 0.001, P = 0.037, and P = 0.03, respectively; R^2^ = 0.65). In the final multivariable model, the diameter of the feeding artery had the strongest correlation with hypoxaemia (R^2^ = 0.43). The diameter of the feeding artery had a significant linear correlation with the oxygen pressure prior to any treatments (P < 0.0001) (Fig. 2C). According to the linear correlative model and the scatter plot graph, the oxygen pressure was < 80 mmHg when the diameter of the feeding artery was > 4.1 mm. The results of the blood gas analysis before and after embolisation were available in 24 patients and showed significant differences (before, 69.08 ± 13.14 mmHg; after, 96.98 ± 25.89 mmHg; P < 0.0001).

**Table 4:** Hierarchical multiple regression analysis of relationship between multiple factors and hypoxaemia

Variables	Model1		Model2		Model3		Model4		Model5		Model6		Model7	
	Coefficients	Standardized Coefficients	Coefficients	Standardized Coefficients	Coefficients	Standardized Coefficients	Coefficients	Standardized Coefficients	Coefficients	Standardized Coefficients	Coefficients	Standardized Coefficients	Coefficients	Standardized Coefficients
Largest diameter of afferent artery (mm)	− 3.64	− 0.66	− 3.37	− 0.61	− 2.39	− 0.43	− 2.62	− 0.47	− 2.63	− 0.48	− 2.64	− 0.48	− 2.51	− 0.45
Largest diameter of the sac (mm)			− 0.07	− 0.09	− 0.09	− 0.12	− 0.04	− 0.05	− 0.03	− 0.04	− 0.03	− 0.04	− 0.08	− 0.11
Type of PAVMs (1/2/3/4)					− 5.59	− 0.42	− 3.26	− 0.25	− 3.29	− 0.25	− 3.26	− 0.25	− 3.92	− 0.3
Number of PAVMs							− 4.75	− 0.25	− 5.16	− 0.27	− 5.14	− 0.27	− 3.09	− 0.16
Age									− 0.05	− 0.06	− 0.04	− 0.05	0.03	0.04
Gender											0.66	0.02	2.18	0.08
Other lung diseases													− 6.05	− 0.2
R^2^	0.43		0.44		0.59		0.62		0.62		0.62		0.65	
F value	46.93		23.59		28.6		22.84		18.96		15.56		14.92	
△R2	0.43		0.01		0.15		0.03		0		0		0.03	
△F value	46.93		0.57		22.21		4.53		0.41		0.08		4.8	
Sig. △F value	< 0.001		0.45		< 0.001		0.037		0.53		0.78		0.03	

Type of PAVMs: 1 for simple type, 2 for complex type, 3 for diffuse type, 4 for mixed types. Other lung diseases: bronchiectasis was present in two patients, lobectomy in two, chronic bronchitis in four, pulmonary fibrosis in one, pneumonectasis in two, tuberculosis in one, and pulmonary arterial hypertension in two. PAVMs, pulmonary arteriovenous malformations.

### Associations between collective characteristics of PAVMs and central nervous system manifestations

The results of the ordered multivariable logistic regression analysis showed no association of the largest diameter of the feeding artery and sac of the PAVMs, the type and number of PAVMs, sex, and age with the severity of central nervous system manifestations (P = 0.8, P = 0.42, P = 0.99, P = 0.35, P = 0.17, and P = 0.68, respectively). These results are shown in Table 5. There were no significant differences in the largest diameter of the feeding artery among patients with different levels of central nervous system symptoms (P = 0.44) (Fig. 2D). The range of the largest diameter of the feeding artery was similar among all levels (lF value = 0.11, P = 0.95) (Fig. 2E).

**Table 5:** Ordered multivariable logistic regression analysis of relationship between multiple factors and central nervous system symptoms

Variables	χ^2^	P value
Largest diameter of afferent artery (mm)	1.01	0.8
Largest diameter of the sac(mm)	4.99	0.42
Number of PAVMs	6.7	0.35
Type of PAVMs	0.32	0.99
Age	3.13	0.68
Gender	1.93	0.17

*PAVMs* pulmonary arteriovenous malformations

### Follow-up

Among 81 patients who received treatment, only 1 patient with a diffuse PAVM developed a relapse 2 days after embolisation and underwent video-assisted thoracoscopic surgery. No PAVMs recurred in the other 80 patients during follow-up. Before embolisation, 46 patients had dyspnoea on exertion, and this symptom was greatly relieved in all 46 patients after embolisation (mean age 50 ± 15.55 years; range 16–76 years). In five patients who underwent thoracoscopic surgery, dyspnoea on exertion persisted because of lung lobe resection. Before embolisation, 17 patients had serious central nervous system manifestations (TIA in 12, stroke in 4, and a brain abscess in 1), and none of these patients developed relapse of symptoms during follow-up (median age 49 years; range 31–64 years).

### Current treatment status

Fifty-nine doctors (48.76%) were unfamiliar with PAVMs and rarely encountered patients with PAVMs. A total of 95.04% of doctors (115/121) knew that most PAVMs are congenital, but 60.37% of doctors (73/121) did not link PAVMs to HHT in clinical practice. Ninety-eight doctors (80.99%) believed that PAVMs do not induce serious manifestations. Most doctors (up to 86.68%) realised the correlation between respiratory symptoms and PAVMs, but only up to 28.93% of doctors (35/121) knew that PAVMs induce central nervous system manifestations. Thirty-eight doctors (31.4%) did not fully understand all the serious complications of PAVMs (Fig. 2F). The doctors were divided on the treatment of PAVMs, and 50.41% of doctors believed that PAVMs do not require medical attention. One hundred eleven doctors (91.74%) considered that embolisation is the best alternative for treatment of PAVMs if necessary, but 64.46% of doctors (78/121) were unaware that the presence of feeding arteries of ≥ 3 mm in diameter is the proper indication for embolisation.

## Discussion

This is the largest-sample study to describe the characteristics of PAVMs in the general population. MSCT and CTA (resolution: 1.0 × 1.0 mm or 0.625 × 0.625 mm respectively) was conducive to the detection of very small PAVMs in our study, and this noninvasive imaging technique can clearly reveal the construction of PAVMs. We recommend that CT plays the largest role in diagnosis of PAVMs [4–6, 23, 24]. The detection rate of PAVMs was 0.026%, which is lower than that reported by Nakayama et al. [6] (0.038%). The hospitals in this survey are top national research-oriented hospitals serving a large nationwide population. Although the present study was not a population study, the detection rate might indicate a low incidence of PAVMs in the general population. In total, 37.7% of the enrolled patients were diagnosed with HHT, which is a lower rate than in Western populations [1, 25]. We considered that the correlation between PAVMs and HHT is indeed much lower in Asian populations, which is in line with previous studies [22, 26]. The present study also indicated that female patients represented the major group of patients with PAVMs [6, 27]. Because increasingly more idiopathic PAVMs are being found in patients without HHT, we consider that PAVM research should be performed in the general population, not just in patients with HHT.

The relationships between the collective characteristics of PAVMs and various clinical symptoms were specifically revealed in this study. Given the influence of multiple factors of PAVMs on hypoxaemia, the largest diameter of the feeding artery (≥ 3 mm) should no longer be the only reference standard for treatment. The number and type of PAVMs should also be taken into consideration. For patients with multiple small PAVMs (< 3 mm diameter of feeding artery), the total right–left shunt could also result in hypoxaemia. In our study, all multiple telangiectatic PAVMs were found in teenagers or young adults diagnosed with HHT, and the growth and development of these patients were affected by hypoxaemia. Complex or diffuse PAVMs are often accompanied by hypoxaemia [28]. Especially in patients with other lung diseases, PAVMs would exacerbate hypoxaemia.

The poor associations between the collective characteristics of PAVMs and central nervous system manifestations indicate that serious symptoms develop because of the existence, not size, of a right–left shunt. A small PAVM (≤ 3-mm diameter of feeding artery) can also result in serious symptoms such as TIA, stroke, or brain abscess [29]. Therefore, treatment is recommended for PAVMs with a small-diameter feeding artery (< 3 mm), which differs from previous studies [14]. In our study, timely treatment of PAVMs reduced the recurrence rate of serious central nervous system manifestations in patients who had once shown such symptoms. However, whether early treatment of small PAVMs decreases the rate of TIA, stroke, and brain abscesses in the younger population remains unknown. This requires further study.

According to our experience, embolisation should still be the first-line treatment. In contrast to thoracoscopic surgery, embolisation eliminates the right–left shunt without impairing lung function. For small, distally located PAVMs, we still consider embolisation to be the first choice, which is inconsistent with a previous study [22]. In our experience, such PAVMs can also be embolised with micro-catheters and micro-coils after careful performance of super-selective angiography. We also consider embolisation to be the first choice for patients with serious haemorrhagic complications because of the high mortality rate of haemorrhagic anaemia and respiratory insufficiency during surgical resection [14, 30–32]. Diffuse PAVMs might recur after embolisation because of untreated feeding arteries, and thoracoscopic surgery should be the priority.

Inquiry into the patients’ first hospital visits in this study revealed that the causes of the hospital visits were quite different and that the distribution of the consulting departments was extremely uneven. However, at least half of the doctors could not give proper treatment recommendations because of their lack of awareness about PAVMs. Therefore, it is necessary to clarify the associations between the collective characteristics of PAVMs and various manifestations and clearly describe the characteristics of PAVMs in the real-world clinical setting. Pulmonologists, thoracic surgeons, and radiologists should be familiar with the diagnosis and treatment of PAVMs.

This study had limitations because of its retrospective nature. The medical records of some patients were limited, and telephone follow-ups could only provide the patients’ medical data; such follow-ups were not helpful for obtaining the family history of HHT. Furthermore, we were unable to perform genetic testing for HHT in all patients. Determination of the accurate incidence of PAVMs in China requires further research given the possible selection bias in the present study.

## Conclusions

The incidence of PAVMs is low in the general population. PAVMs are much less associated with HHT than previous reported. MSCT and CTA should be conventionally used for diagnosis of PAVMs. Given the connection between the collective characteristics of PAVMs and various manifestations, the number and type of PAVMs should be taken into consideration in addition to the diameter of the feeding artery. Small PAVMs should be treated in patients who develop serious manifestations. Transvascular embolisation should still be the first treatment choice.

## Data Availability

The datasets used and/or analysed during the current study are available from the corresponding author on reasonable request.

## Abbreviations

PAVMs: Pulmonary arteriovenous malformations
HHT: Hereditary hemorrhagic telangiectasia
MSCT: Multi-slice spiral computed tomography
CTA: Computed tomography angiography
TIA: Transient ischaemic attack
